# New versus Old Oral Anticoagulants: How Can We Set the Scale Needle? Considerations on a Case Report

**DOI:** 10.3390/medicina55030071

**Published:** 2019-03-17

**Authors:** Francesca Antonia Arcadi, Simona Portaro, Roberto Giorgianni, Antonino Naro, Carmela Casella, Carmelo Genovese, Silvia Marino, Rocco Salvatore Calabrò

**Affiliations:** 1 IRCCS Centro Neurolesi “Bonino-Pulejo”, 98123 Messina, Italy; francesca.arcadi@irccsme.it (F.A.A.); simona.portaro@irccsme.it (S.P.); roberto.giorgianni@irccsme.it (R.G.); antonino.naro@irccsme.it (A.N.); silvia.marino@irccsme.it (S.M.); 2 Stroke Unit, Policlinico Universitario, 98123 Messina, Italy; ccasella@unime.it; 3 Genovese Laboratory Medicine and Pathology, 98050 Barcellona PG, Italy; info@laboratoriogenovese.it

**Keywords:** dabigatran, warfarin, ischemic stroke, anticoagulants

## Abstract

Ischemic stroke is a complex multifactorial disorder. Anticoagulation is a growing research area, with the main goal of preventing systemic embolization and stroke. We report the case of a 41-year-old woman with antiphospholipid syndrome who was unsuccessfully treated with Dabigatran, a new oral anticoagulant, as she developed a major stroke involving the right carotid artery, due to deep venous thrombosis with pulmonary embolism. We therefore suggest a closer monitoring of the safety and efficacy of dabigatran. Moreover, in the presence of multifactorial causes of pro-coagulation, we believe that warfarin should remain the mainstay of oral anticoagulation.

## 1. Introduction

Anti-phospholipid syndrome (APLS) is a systemic autoimmune disease, characterized by venous or arterial thrombosis and/or poor obstetrical outcomes, and associated with raised plasma levels of anti-phospholipid antibodies (aPL) [1,2]. APLS can be isolated, as with the Hughes syndrome [1], or occur within other autoimmune disorders. The mechanisms by which aPL causes thrombosis are not completely understood [1].

The physiological coagulation cascade is the process through which blood clots are rapidly formed to arrest hemorrhage once blood vessel injury occurs. The fibrinolytic system occurs in order to degrade the blood clots, avoiding the obstruction of the blood flow. Indeed, the coagulation process quickly generates thrombin, also known as factor IIa (FIIa), the enzyme that converts fibrinogen to fibrin and serves as a potent platelet agonist [3,4,5,6,7,8]. Antithrombotic drugs are applied to destroy two different types of thrombi: the ones located in the venous system, made by fibrin, platelets and red blood cells, and those located in the arterial system consisting of a larger amount of platelets with less fibrin. Thus, medications affecting coagulation act on specific sites of these thrombi. Specifically, antiplatelet drugs (i.e., aspirin and clopidogrel) and fibrinolytics (streptokinase and alteplase) target arterial thrombi, whereas traditional anticoagulants (i.e., heparin, low-molecular-weight (LMW) heparins and fondaparinux), vitamin K antagonists (VKAs) (warfarin), and direct-acting oral anticoagulants (DOACs) (dabigatran, rivaroxaban and apixaban) target venous or stasis-induced thrombi [9]. The main concern in APLS management includes the treatment of acute thromboembolic manifestations, the choice and duration of anticoagulation, and the first thrombosis prevention. Aspirin is not considered the drug of choice for APLS, which is frequently treated with anti-vitamin K anti-coagulants [10]. However, whether these patients should receive oral anticoagulation (either vitamin K antagonists or one of the new oral anticoagulants) or drugs that target one or more of the possible pathogenic mechanisms of thrombosis is still under debate.

We report the case of a 41-year-old woman with antiphospholipid syndrome unsuccessfully treated with Dabigatran, a DOAC, as she developed a major stroke involving the right carotid artery, due to deep venous thrombosis with pulmonary embolism.

## 2. Case Presentation

A 41-year-old woman came under our observation to undergo intensive neurorehabilitation due to ischemic stroke. Her family history was negative for neurological disorders. Her personal history was unremarkable, and neither smoking habits nor alcohol or drug consumption were reported. She denied the use of oral contraceptives or other drugs potentially affecting coagulation. Body mass index was within the normal range (a BMI of 23). She had a personal history of migraine, high blood pressure, and nodular thyroid disease. After one month from a miscarriage with intrauterine death of the fetus (at the 26th week of gestation), she presented a thrombosis of the left popliteal vein with pulmonary embolism. A treatment with dabigatran (150 mg/twice a day) was prescribed. One month later, she suddenly presented with difficulty in moving her right limbs and in articulating words. She was then admitted to a Stroke Unit. Neurological examination showed a right deviation of head and eyes, and a left hemiplegia with homolateral dysesthesias (NIH-Stroke Scale score: 15). She then underwent a computed tomography angiography, detecting a right M2 occlusion, with a consequent mechanical thrombectomy. During admission, she was submitted to several investigations, including (i) chemiluminescent immunoassay (CLIA) for the detection of anticardiolipin antibodies (aCL) and enzyme-linked immunosorbent assay (ELISA) for the IgM/IgG anti-b2 glycoprotein I; (ii) functional clotting time-based assay for the determination of the lupus anticoagulant; (iii) transcranial Doppler with microbubble test; and (iv) trans-esophageal Doppler. The immunological tests were performed using the LIAISON^®^ Cardiolipin IgM/IgG CLIA assay and the ETI-Beta 2 Glycoprotein I IgM/IgG ELISA kit (DiaSorin; Sallugia, Italy). The immunological tests were performed using the LA1 Screening Test and LA2 Confirm test by Sysmex South Africa (Pty) Ltd. (Ferndale, Randburg; South Africa). 

Specifically, there were high levels of aCL (IgG 764.1 CU—n.v. < 20; IgM 167.90 CU—n.v. < 20), whereas the IgG/IgM antib2-glycoprotein I and lupus anticoagulant were within the normal range. The transcranial Doppler with microbubble test disclosed a right-to-left shunt with a bubble passage > 25 at rest. Finally, the trans-esophageal Doppler showed a patent foramen ovale (2.5 mm × 5 mm). She was therefore switched from dabigatran to acenocumarole (4 mg/daily). At discharge, she presented amaurosis in the right eye, distal weakness at the left upper limb and a left tendon hyperreflexia, with an NIHSS of 3. At one-year follow-up, after a 3-month-rehabilitation, clinical conditions further improved without any sign/symptom of thromboembolism.

The patient gave written consent for publication of the case.

## 3. Discussion

Ischemic stroke is a complex multifactorial disorder and it is considered the main cause of disability among the elderly. Patent foramen ovale, paradoxical embolism from peripheral venous system, embolization from thrombi formed within the atrial septum, intracardiac thrombi as a result of arrhythmias, and inherited and secondary thrombophilia are considered important stroke risk factors [11]. However, there are other uncommon causes of thrombosis [12,13]. Specifically, it is known that aPL is causatively related to the clinical manifestations of APLS, and are not merely innocent markers of aPL related ischemic stroke [1,2].

In order to prevent such events, a great deal of progress has been made over the past decade in pharmacological research. Anticoagulation is an evolving research area, with the main goal of preventing systemic embolization and stroke. Which drug is better in the treatment of patients with thrombosis is still under debate. In fact, there are no substantive data to support the use of the DOACs over warfarin. The Randomized Evaluation of Long-Term Anticoagulation Therapy trial, performed in patients with atrial fibrillation, found that hemorrhagic strokes were significantly reduced in the group receiving dabigatran than in those receiving warfarin [14]. However, this does not elucidate the role of DOAC therapy in preventing thrombi formation [15]. Considering patients with acute venous thromboembolism (VTE), including those with APLS, two main clinical trials, i.e., the RE-MEDY and the RE-COVER trials, should be taken into account. In these interesting studies, it has been demonstrated that dabigatran had similar effects on VTE recurrence and a lower risk of bleeding when compared to warfarin [16,17]. Moreover, it has been suggested that dabigatran may be useful in the treatment of APLS, because of its safety [18,19]. Nonetheless, the efficacy of dabigatran in stroke treatment came from single and conflicting case reports, some supporting [20,21] and others arguing against the beneficial effects of the drug [22,23,24]. From these data, it has been shown that dabigatran non-significantly reduced patients’ hazard of developing ischemic stroke, as also reported from recent reviews [25]. Thus, it is plausible that dabigatran may not significantly reduce ischemic stroke, as compared to warfarin [25].

Warfarin (an old anticoagulant) is a vitamin K antagonist, which blocks the γ-carboxylation of glutamate residues and inhibits vitamin K reductase. This process influences the coagulation cascade interfering with the vitamin K-dependent clotting factors (II, VII, IX, X) synthesis, since carboxylation is required for their conformational change [26].

A patient’s prothrombin time (PT), standardized by the international normalized ratio (INR) test, is the tool for clinicians to monitor the intensity of coagulation, with a recommended mean therapeutic range between 2.0–3.0 [27]. Apart from its low cost, warfarin is safe in thrombosis prevention, despite the need to constantly monitor the INR and the known interaction with several foods, such as green leafy vegetables, which have high vitamin K levels. Moreover, even medications such as antibiotics, antifungal medication, naproxen sodium, etc. may represent a concern for its use. Thus, initial INR monitoring should be performed daily for inpatients and every two/three days for outpatients, until the INR is stable. Then, an interval not exceeding 4 weeks between monitoring is required [27,28]. 

On the other hand, DOACs, such as dabigatran, which were first designed to face the limitations related to warfarin use, have distinct advantages over warfarin and, consequently, their availability is increasing. In fact, dabigatran is a reversible direct thrombin inhibitor that prevents the conversion of fibrinogen to fibrin, thus leaving a small amount of free active thrombin for haemostasis [29]. The main advantage is that it does not need routine monitoring as the old anticoagulants do, because of the non-linear correlation between dabigatran concentration and Activated Partial Thromboplastin Time (aPTT) levels, even if debated during the last years and only recently confirmed [30,31]. As dabigatran is renally excreted, some authors recommend drug monitoring in patients with renal impairment [32].

Dabigatran was ineffective in our patient, maybe due to the complexity of her hypercoagulability state. In fact, the additional effects of several risk factors, including high blood pressure, the increased levels of anticardiolipin and antiphospholipid antibodies and the cohexistence of a patent foramen ovale, may have contributed in making the drug ineffective.

More recently, other DOACs, such as rivaroxaban, have been shown not to be indicated for APS. In fact, rivaroxaban was also associated to an increased rate of events compared with warfarin, thus not being indicated to treat patients with APS [33].

## 4. Conclusions

Even though our findings are based on a single case report, we suggest a closer monitoring of the efficacy and safety of dabigatran, and a drug surveillance investigation on a larger similar case series. In fact, in the presence of multifactorial causes of pro-coagulation, warfarin remains the mainstay of oral anticoagulation. Indeed, we recommend warfarin to treat patients at high thrombophilic and thrombotic risk, mainly because of its four distinct advantages over DOACs: (i) no dose adjustment is required, even in cases of renal failure; (ii) therapeutic monitoring is readily accessible; (iii) the antidote is readily available; and (iv) its better efficacy in managing thrombotic complications.
